# Roadmap for Children’s Health: Controlling Diverse Environmental Exposures in Latin America

**DOI:** 10.1289/ehp.123-A70

**Published:** 2015-03-01

**Authors:** Carol Potera

**Affiliations:** Carol Potera, based in Montana, also writes for *Microb*e, *Genetic Engineering News*, and the *American Journal of Nursing*.

Patterns of disease change as countries become industrialized. Whereas the main environmental threats in underdeveloped countries include poorly ventilated indoor cook stoves and contaminated drinking water, more developed countries face exposures related to modern-day life, such as industrial pollution, synthetic chemicals, and hazardous waste.^1^ A 2012 conference convened in Montevideo, Uruguay, by the World Health Organization (WHO) and the Pan American Health Organization (PAHO) focused on the health consequences of environmental exposures in Latin America, a region where both traditional and modern threats exist side by side.^2^ In this issue of *EHP*, participants from that conference review the specific risks to children in this region and strategies for mitigating them.

Indoor household air pollution from solid fuel use has been identified by the WHO and PAHO as a leading environmental health threat in the region, causing an estimated 287,900 deaths in Latin American children under age 5 in 2012.^3^ Lead pollution is widespread in Latin America from battery production and recycling, smelters, paint, and poorly controlled recycling of discarded electronics (e-waste).^4^^,^^5^ Yet there are no uniform epidemiological databases that track children’s blood lead levels in Latin America.^1^ Programs are needed to screen children at risk and to diagnose and treat lead poisoning, says senior review author Philip J. Landrigan, Dean for Global Health at Mount Sinai Hospital in New York City.

**Figure:**
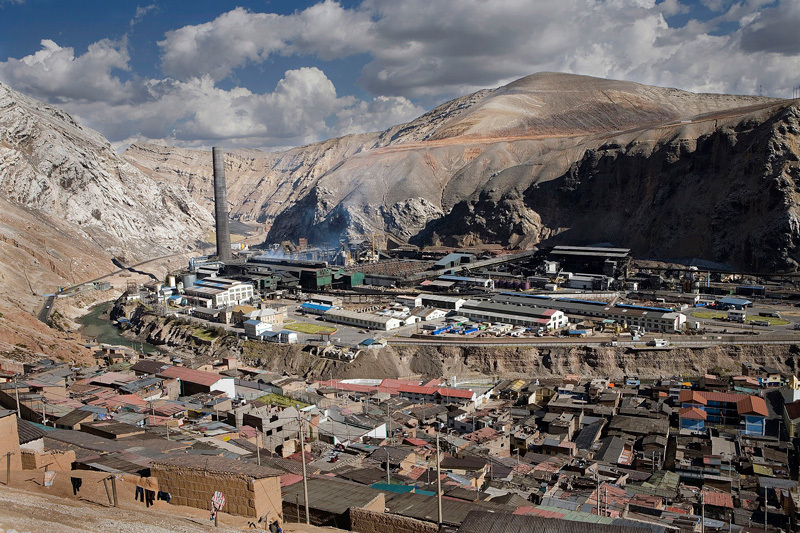
Smelters like this complex in La Oroya, Peru, have contributed to widespread metal pollution in Latin America. © Fernando Moleres/Panos Pictures

Other common exposures include pesticides,^4^ naturally occurring arsenic in drinking water,^6^ and mercury from the artisanal gold mining that is practiced in many parts of Latin America.^7^ Some Latin American countries import e-waste to recover copper and gold—a valuable industry—and the disassembled electronics are a source of exposure to chromium, nickel, manganese, polychlorinated biphenyls, brominated flame retardants, and other toxic chemicals for children living near recycling sites.^8^

The extent of these and other environmental problems must first be identified. Then they can be tackled with programs such as emissions tracking, maps that identify hazardous waste sites, and surveillance systems to monitor the number of cases of asthma, diabetes, cancer, and other illnesses that may be related to environmental exposures.^1^ “Half the battle of controlling any pollutant is being able to measure it,” says Landrigan.

The review authors identified indoor and outdoor air pollution, water pollution, and toxic chemical hazards as priority health areas for Latin America.^1^ In addition to monitoring, the authors recommend studies on the economic costs of environmentally induced disease as well as biomedical research to serve as a basis for evidence-based treatment and prevention.^1^ Landrigan suggests the WHO, the U.S. Agency for International Development, and the National Institute for Environmental Health Sciences (NIEHS) as possible funders for monitoring programs.

Economic data can support the development of health-protective policies.^1^ However, each country must regulate pollutants itself. “WHO and other organizations can only advise countries and set international guidelines,” Landrigan says.

“[The review] presents a roadmap for mitigating or eliminating environmental exposures on children’s health in Latin America,” says David Christiani at the Harvard Schools of Medicine and Public Health, who was not involved in the review. “Evidence-based intervention is necessary, and assembling scientific evidence to guide action is urgently needed. Although progress will be incremental, it’s important to implement these recommendations now.”

“Although the environmental health threats to the children of the Latin America region are not unlike those of children in the rest of the world, their unique circumstances need to be addressed using rigorous scientific and prevention strategies,” says review coauthor William A. Suk, chief of the NIEHS Hazardous Substances Research Branch.

Landrigan says some countries in Latin America have become world leaders in developing strategies to address emerging issues in children’s environmental health. Pediatricians and health scientists in Latin America have built research programs in children’s environmental health, created a network of clinical centers known as Unidades Pediátricas Ambientales (Pediatric Environmental Units) to evaluate children exposed to environmental hazards, developed new educational materials, and issued declarations that affirm the importance of making the protection of children’s health the centerpiece of regional environmental policy. A Spanish-language translation of the new review will be used as an educational tool in Latin America by WHO Collaborating Centers and the Unidades Pediátricas Ambientales.
